# Fortification of Human Milk for Preterm Infants: Update and Recommendations of the European Milk Bank Association (EMBA) Working Group on Human Milk Fortification

**DOI:** 10.3389/fped.2019.00076

**Published:** 2019-03-22

**Authors:** Sertac Arslanoglu, Clair-Yves Boquien, Caroline King, Delphine Lamireau, Paola Tonetto, Debbie Barnett, Enrico Bertino, Antoni Gaya, Corinna Gebauer, Anne Grovslien, Guido E. Moro, Gillian Weaver, Aleksandra Maria Wesolowska, Jean-Charles Picaud

**Affiliations:** ^1^Division of Neonatology, Department of Pediatrics, Istanbul Medeniyet University, Istanbul, Turkey; ^2^PhAN, Institut National de la Recherche Agronomique (INRA), Université de Nantes, CRNH-Ouest, Nantes, France; ^3^Department of Nutrition and Dietetics, Imperial College Healthcare NHS Trust, London, United Kingdom; ^4^Lactariums de Bordeaux-Marmande, Pôle Pédiatrique, Centre Hospitalo-Universitaire (CHU) de Bordeaux, Bordeaux, France; ^5^Neonatal Unit of Turin University, City of Health and Science of Turin, Turin, Italy; ^6^Greater Glasgow and Clyde Donor Milk Bank, Royal Hospital for Sick Children, Glasgow, United Kingdom; ^7^Banc de Teixits, Fundaciò Banc Sang i Teixits de les Illes Balears, Palma de Mallorca, Spain; ^8^Abteilung Neonatologie Klinik und Poliklinik für Kinder und Jugendliche, Leipzig, Germany; ^9^Neonatal Unit, Milk Bank, Oslo University Hospital, Oslo, Norway; ^10^Associazione Italiana Banche del Latte Umano Donato (AIBLUD), Milan, Italy; ^11^Hearts Milk Bank, Rothamsted Research Institute, Harpenden, United Kingdom; ^12^Department of Neonatology, Faculty of Health Science, Medical University of Warsaw, Warsaw, Poland; ^13^CarMeN Unit, INSERM U1060, INRA U1397, Claude Bernard University Lyon 1, Pierre Bénite, France; ^14^Division of Neonatology, Hôpital de la Croix-Rousse, Lyon, France

**Keywords:** nutrition, prematurity, human milk, adjustable fortification, individualized fortification, growth, protein

## Abstract

Evidence indicates that human milk (HM) is the best form of nutrition uniquely suited not only to term but also to preterm infants conferring health benefits in both the short and long-term. However, HM does not provide sufficient nutrition for the very low birth weight (VLBW) infant when fed at the usual feeding volumes leading to slow growth with the risk of neurocognitive impairment and other poor health outcomes such as retinopathy and bronchopulmonary dysplasia. HM should be supplemented (fortified) with the nutrients in short supply, particularly with protein, calcium, and phosphate to meet the high requirements of this group of babies. In this paper the European Milk Bank Association (EMBA) Working Group on HM Fortification discusses the existing evidence in this field, gives an overview of different fortification approaches and definitions, outlines the gaps in knowledge and gives recommendations for practice and suggestions for future research. EMBA recognizes that “Standard Fortification,” which is currently the most utilized regimen in neonatal intensive care units, still falls short in supplying sufficient protein for some VLBW infants. EMBA encourages the use of “Individualized Fortification” to optimize nutrient intake. “Adjustable Fortification” and “Targeted Fortification” are 2 methods of individualized fortification. The quality and source of human milk fortifiers constitute another important topic. There is work looking at human milk derived fortifiers, but it is still too early to draw precise conclusions about their use. The pros and cons are discussed in this Commentary in addition to the evidence around use of fortifiers post discharge.

## Introduction

Inadequate nutrition during the critical periods of brain development alters the growth trajectory of the brain and can have permanent negative consequences. The most critical period of brain growth and development for humans corresponds to the third trimester of pregnancy and for very low birthweight (VLBW) infants these developmental processes take place in the neonatal intensive care unit (NICU) environment (1, 2). Inadequate nutrition and/or poor postnatal growth during the NICU stay has been associated with neurocognitive impairments (3–11) and poor renal function (12) in preterm infants. Recent studies suggest that not only the growth *per se*, but also the quality of growth counts. Better linear growth and early gains in fat-free body mass have been found to be associated with improved neurodevelopment in VLBW preterm infants (13, 14). Thus, optimization of the nutritional care for the preterm infants has a key role in improving neurodevelopmental outcomes and has become a priority.

Despite the advancements in nutritional support over 20 years and current focus on “early intense nutrition” in NICU, undernutrition and extrauterine growth restriction (EUGR) are still important problems for VLBW infants (15–18).

Evidence indicates that human milk (HM) is the best source of nutrition for both term and preterm infants conferring health benefits both in the short and long-term (19, 20). Unfortified HM however does not provide sufficient nutrition to VLBW infants when fed at the usual feeding volumes. Human milk should be supplemented (fortified) with the nutrients in short supply, particularly with protein, calcium, and phosphate to meet high requirements of this group of tiny preterm infants as discussed in the next sections. Although HM fortification is widely adopted in the NICUs all over the world, there is still much inconsistency and variability and even some skepticism around this practice. During the last decade optimization of HM fortification- mainly individualization, and the quality of the fortifiers have been the hot topics of discussion.

The European Milk Bank Association (EMBA) Working Group on HM Fortification aims to document the existing evidence on this field, overviews different fortification approaches, clarifies the terminology and definitions, outlines the gaps in knowledge, and gives recommendations for practice and suggestions for future research.

## Methods

European Milk Bank Association (EMBA) Working Group (WG) on HM Fortification was formed by a group of experts on this field in 2013. In 2016 WG planned to review the related research and to write a position paper with recommendations on HM fortification for preterm infants. The first face-to-face meeting in Milan resulted in organizing the paper into 10 different sections. These sections were then assigned to working subgroups within the WG. The literature review included electronic searches of MEDLINE (1966-30 June 2018), EMBASE (1980-30 June 2018), CINAHL (1981-30 June 2018), the Cochrane Library, and conference proceedings. The electronic search used the following key words: human milk fortification, breast milk fortification, donor milk fortification, banked milk fortification, [human milk OR breast milk] AND [fortification]. All types of articles, including original papers, reviews, and recommendations were considered. Furthermore, the reference lists of the previous reviews and relevant studies were examined. The searches were limited to human studies, and to the published articles written in English. Trials that had been reported only as abstracts were eligible for inclusion if sufficient information was available from the report.

Following the first meeting, a total of 4 face-to-face meetings were held in Milan, Lyon, and Glasgow to formulate and agree on all of the recommendations. All group members interacted during these face-to-face meetings, and by iterative e-mails between them. All conclusions and recommendations were discussed until a full consensus was achieved for each statement.

## The Rationale For Human Milk Feeding And Human Milk Fortification

### Human Milk as the Best Feeding Option for Preterm Infants

Evidence-based data show that HM is the best nutritional and normative standard for infant nutrition (19, 20). Its particular composition—“nutrients with optimal bioavailability, hormonal and enzymatic components, anti-infective, trophic and growth factors, stem cells, prebiotics and probiotics and a myriad of bioactive proteins” -makes HM suited not only to term but also to preterm infants (21–26). Feeding preterm infants with HM, indeed, confers protection against the most important NICU challenges such as necrotizing enterocolitis (NEC) and sepsis (27–33), retinopathy of prematurity (ROP) (34–36), bronchopulmonary dysplasia (BPD) (37, 38) and decreases mortality in a dose-dependent manner (31). Human milk feeding improves long-term neurocognitive development (39–41) and cardiovascular health outcomes (29). Studies comparing solely donor human milk vs. formula show that donor human milk confers protection against NEC (27, 29) and improves feeding tolerance (29). That is why HM is the recommended feeding for all neonates including premature infants. The European Society for Pediatric Gastroenterology Hepatology and Nutrition (ESPGHAN) (29), American Academy of Pediatrics (AAP) (19), and Milan EMBA/ESPGHAN/AAP Joint Meeting Consensus (42) in their most recent recommendation papers stated that “mother's own milk (MOM) is the first choice in the feeding of preterm infants. When mother's milk is not available, pasteurized donor human milk (DHM) should be used.”

### The Rationale for Human Milk Fortification

Infants born early in the third trimester miss the placental transfer of nutrients which would create stores for use in the postnatal period. Human milk while acting as “a preventive therapeutic drug,” doesn't provide sufficient amounts of many nutrients for premature infants when fed at the usual feeding volumes. The main challenge is to meet the high and variable nutrient requirements of these preterm infants during the whole hospitalization period. Insufficient nutrient intakes place the infant at risk of impaired neurodevelopment. To prevent EUGR, which is associated with poor neurocognitive outcome, and to avoid specific nutrient deficiencies, nutrient fortification of HM is necessary (19, 29, 42–46).

The consequences of intakes falling short of requirements vary from nutrient to nutrient. Evidence suggests that inadequate intake of protein is important for slow growth and it is particularly responsible for decreased fat-free mass (FFM) gains which are directly related to poor neurocognitive outcomes (14, 47). Intake of energy is also clearly important. In a single blinded randomized clinical trial, Bellagamba et al. (48) showed that increasing only protein intake by 1 gram during parenteral and enteral nutrition did not improve growth and neurodevelopment of preterm infants with a birthweight 500–1,249 g. Insufficient intake of some nutrients leads to specific deficiency states, such as osteopenia (due to insufficient intake of calcium and phosphorus) and to various micronutrient deficiencies, such as zinc deficiency. It is important that VLBW infants receive adequate amounts of iron, zinc, copper, selenium, and iodine. The need of fortification is less clear with regard to manganese, chromium, and molybdenum (49). For the great majority of other nutrients, small shortfalls may have less serious effects, especially when they are temporary. With protein however, any shortfall is prone to affect growth and carries the risk of neurocognitive impairment. Thus, protein supply needs special attention in early life and meeting the requirements should be the goal (43, 44).

*The objective of fortification* is to increase the concentration of nutrients to the levels that at the recommended feeding volumes (135–200 ml/kg/d) preterm infants receive amounts of all nutrients that meet requirements (43, 50). Nutrient requirements of preterm infants are defined as intakes that enable the infant to grow at the same rate as a fetus (44). Requirements for most nutrients have been derived from accretion rates of protein, fat and minerals obtained by the analysis of fetal body composition at various stages of gestation (44, 50, 51). Additionally, empirical methods have been employed to define requirements including those for nutrients such as vitamins (44, 51) (Tables 1, 2). However, these requirements are variable depending on the clinical condition and characteristics of each infant either present at birth or evolving during NICU stay (such as IUGR or severe BPD). Therefore, HM fortification needs to be adapted to the specific needs of each infant at each time.

**Table 1:** Requirements for protein and energy; best estimates by factorial and empirical methods (44).

**Body weight, g**	**500–1,000**	**1,001–1,500**	**1,501–2,000**
Weight gain of fetus, g/kg/d	19.0	17.4	16.4
Protein, g/kg/d	4.0	3.9	3.7
Energy, Kcal/kg/d	106	115	123
Protein/energy, g/100 kcal	3.8	3.4	3.0

**Table 2:** Requirements for major minerals and electrolytes determined by factorial method, listed by body weight (51).

	**500–1,000 g**		**1,001–1,500 g**		**1,501–2,000 g**	
	**Accretion**	**Requirem**.	**Accretion**	**Requirem**.	**Accretion**	**Requirem**.
Ca (mg)	102	184	99	178	96	173
P (mg)	66	126	65	124	63	120
Mg (mg)	2.8	6.9	2.7	6.7	2.5	6.4
Na (meq)	1.54	3.3	1.37	3.0	1.06	2.6
K (meq)	0.78	2.4	0.72	2.3	0.63	2.2
Cl (meq)	1.26	2.8	0.99	2.7	0.74	2.5

The EMBA Working Group on HM Fortification summarizes the latest recommended intakes for protein, carbohydrates, lipids, and energy in Table 3. This table comprises the recommendations of the experts and expert panels (50, 52, 53).

**Table 3:** Recommended enteral protein and energy intakes for clinically stable very low birthweight infants (50, 52, 53).

	**Munich consensus 2014**	**ESPGHAN 2010**	**Ziegler et al**.
Energy (kcal/kg/d)	110–130	110–135	105-127
Protein (g/kg/d)	3.5–4.5	4.0–4.5 (<1 kg) 3.5–4.0 (1–1.8 kg)	3.9-4.0
Protein/Energy (g/100 kcal)	3.2–4.1	3.2–4.1	3.1–3.8
Lipids (g/kg/d)	4.8–6.6	4.8–6.6	–
Carbohydrates (g/kg/d)	11.6–13.2	11.6–13.2	–

## Current Human Milk Fortifiers And Supplements

There are a number of products available for fortifying human milk for preterm babies which differ by the origin of milk used (bovine, human or donkey), and by nutrient composition (multi-nutrient fortifiers or supplements of protein, lipids, carbohydrates).

### Multi-Nutrient Fortifiers

Bovine-based multi-nutrient fortifiers contain varying amounts of protein, energy, minerals, trace-elements, vitamins, and electrolytes (Table 4). The addition of lipids to multi-nutrient fortifiers with a concomitant reduction in carbohydrate content has allowed a reduction in osmolality of these products (54). In addition, lipids provide a source of essential fatty acids (EFA) which has been shown to improve EFA status in preterm infants (55). As indicated, standard fortification using previously available products was unable to support a satisfactory postnatal growth (See Current Fortification Practices in Neonatal Intensive Care Units: Terminology-Definitions). New fortifiers with higher protein content have been shown to improve short term weight gain (56). Most multi-nutrient fortifiers contain bovine milk protein. Donkey milk was more recently proposed as its composition is very close to human milk (57).

**Table 4:** Nutrient composition of selected fortifiers and supplements.

		**Bovine-based products (per gram of powder)**									**Human milk-based fortifier (per volume)**			
	**Multicomponent fortifiers**					**Protein supplements**								
Fortifier	A	B	C	D	E	F	G	H	I	J	K	L	M	N
Volume (ml)	/	/	/	/	/	/	/	/	/	/	20	30	40	50
Energy (kcal)	4.4 (L)	3.5	3.6	4.9 (L)	3.9 (L)	3.4	3.6	3.6	4	3.7	28	42	56	71
Protein (g)	0.36^PH^	0,25^EH^	0.2^EH^	0.4	0.3	0.82^EH^	0.72^EH^	0.86^W^	0.8^W^	0.9^W^	1.2	1.8	2.4	3
Na (mg)	9.2	8,0	5.4	5.6	4.2	7.8	8.2	2.1	2	0	20	40	42	45
Ca (mg)	18.9	14.9	10	32	33	5.2	12.8	0	4	0	103	106	108	111
P (mg)	11	8.7	7	18	19	5.2	0.73	0	3	0	53.8	54.9	56	57.5
Iron (mg)	0.5	0	0	0.5	0.1	0	0.007	0	0	0	0.1	0.15	0.2	0.25

*L, lipids; PH, partially hydrolyzed; EH, extensively hydrolyzed; W, whole protein; HMBF, human milk-based fortifier. A-Fortipré®, Nestle; B-Fortema®, Danone; C-FM85®, Nestle; D-Enfamil®, Mead Johnson; E-Similac®, Ross; F-Aptamil PS®, Danone; G-Preemie®, Nestle; H-Beneprotein®, Nestle; I-Pro-Mix®, Corpak; J-Protein instant®, Resource; K- HMBF+4®, Prolacta; L- HMBF+6®, Prolacta; M- HMBF+8®, Prolacta; N- HMBF+10®, Prolacta*.

During the past 15 years, some for-profit companies have been set up to collect and buy HM, to manufacture and to sell HM-based products. Prolacta Bioscience is the only one which produces pasteurized HM and HM-based fortifiers. They adhere to the Human Milk Banking Association of North America (HMBANA) guidelines, but test also bacterial content before heat treatment (including pathogens, such as *Bacillus cereus, Escherichia. coli, Pseudomonas aeruginosa, Salmonella spp*., yeast and mold), recreational drugs, nicotine, prescription drugs, milk adulteration and breast milk DNA fingerprint for donor identification. To treat huge volumes of HM (1,200 L from 250 donors) they use Vat pasteurization (63°C, ≥ 30 min). Vat differs from Holder pasteurization which is the commonly used method in non-profit HM banks. Meredith-Dennis et al. (58) showed that Vat pasteurization significantly reduced lactoferrin and total HM oligosaccharide concentrations when compared to Holder pasteurization. Human milk-based fortifier is obtained by concentrating heat-treated donor HM and then adding vitamins and minerals. Various caloric densities of this fortifier allow for individual adjustment based on growth or blood urea nitrogen (BUN). More recently, a novel HM derived cream supplement has been produced by the same company (59, 60).

Although some studies suggested a benefit in terms of morbidity and mortality when babies are fed an exclusively human milk based diet including HM-based fortifier, leading to a reduction of costs (33, 61), much of the work is observational (62–64), and there are still concerns about the efficacy of these products (65). For example, Sullivan et al. (33) showed a significant reduction in NEC rates from 16 to 6%, but this needs to be confirmed in large, independent randomized control trials conducted in units where baseline NEC rates are lower. Sullivan et al. evaluated an exclusive HM-based diet, which consisted of donor HM if no mother's own milk was available and a HM–based fortifier in place of bovine-based formulas and fortifiers. However, the HM-based fortifier was never directly compared with the bovine based fortifier and many of the babies who developed NEC on the bovine fortifier were also on the bovine formula. The OptiMoM study, recently published by O'Connor et al. (66), is the first trial comparing the efficacy of HM-based fortifier to bovine-based fortifier in the absence of formula. There was no difference in feeding tolerance, postnatal growth and morbidity, including NEC ≥ grade 2 (4.7 vs. 4.9%). In 2015, most facilities in US fortified human milk, and approximately one out of five used a HM-based fortifier (67). In summary, HM-based products have been adopted in neonatal care despite being costly and supported by limited efficacy data. Some aspects have not been fully investigated yet, such as metabolic effects and body composition, which are needed before considering these products to be totally safe and effective. It is essential to evaluate the benefit-risk ratio, particularly as these products are very expensive and use large amounts of donated milk to make the fortifier which could be used more directly to feed preterm babies. At the present time these products are available mainly in North America. According to regulations in some European countries, only HM banks in each country are authorized to collect, treat and distribute HM or HM-based products (68, 69). Finally, there could be some ethical concerns. According to available information ethical concerns seem to be well-controlled by present manufacturers but, if the evidence confirms a benefit, the need for these products could increase sharply and ethical questions related to the origin of HM could become a major concern.

In some fortifiers, manufacturers used a hydrolyzed protein source (Table 4). There is no evidence supporting the use of such a protein source. It has been shown that preterm infants fed a formula with partially hydrolyzed protein have a shorter transit time, but also a reduced intestinal absorption (70). The rationale cannot be related to the hypothetical prevention of allergy. Indeed, no increased risk of allergy was detected with preterm infants fed on formulas based on cow's milk even those with a high protein content. It has even been suggested that preterm birth reduces the chances of the subsequent development of severe atopic disease (71). Nevertheless, the use of a hydrolyzed protein source is a response to clinicians' preferences, as a lot of professionals are reluctant to add whole bovine protein to HM. This current opinion of professionals comes from a study suggesting that, in a subgroup of preterm infants with a family history of atopy, early exposure to cow's milk increased the risk of allergic reaction (72). However, more recent studies showed that, compared to exclusively breastfed, preterm infants supplemented with HMF or fed exclusively a preterm formula for 4 months after discharge did not have an increased risk of developing allergic diseases during the first year of life (73). Furthermore, it was previously shown that protein supplementation using whole-protein is efficient (43, 74, 75). In summary, there is no strong evidence to support the use of hydrolyzed protein source in fortifiers, but it is current practice.

### Single-Nutrient Supplements

Other products containing only protein, lipids, or carbohydrates are also available. They are useful when individualizing fortification (74–76). Usually, carbohydrate supplements are composed of dextrin maltose, and lipids are composed of medium chain triglycerides. More recently, a novel HM-derived cream supplement has been produced to enhance the energy density of feeds. Infants were supplemented with the 2.5 kcal/ml cream supplement whenever their mother's own milk or donor HM was found to be below 67 kcal/dl (20 kcal/oz) (60). When compared to the control group these infants had improved weight and length growth rates and were discharged slightly earlier. This reduction in length of stay was greater in the subgroup of preterm infants with bronchopulmonary dysplasia (59, 60). However, this finding needs to be replicated in other settings to ensure that this can be done without compromising protein to energy ratio.

Protein supplements have been available for years in some countries, but are not specifically designed for neonates (74–77). One of them contained extensively hydrolyzed protein source (56). Recently a new protein supplement—including partially hydrolyzed protein source–specifically designed for preterm infants, became available in most European countries (54) (Product G, Table 4). There is no consensus about how to use these products as studies are scarce. That being said, protein supplements are essential to enable individualized fortification, particularly for Adjustable (ADJ) fortification which has been shown to be associated with clinical benefits (74) (see Individualized Fortification).

## Current Fortification Practices In Neonatal Intensive Care Units: Terminology-Definitions

Following the first introduction of the commercial HM fortifiers in the 1980s, HM fortification has become part of the standard nutritional care for preterm infants in most NICUs. The quality of the fortifiers and the methods of HM fortification have improved over time but nutrient fortification remains suboptimal. An optimal approach to fortification is to provide each individual baby with her/his needs, which might be different from the average of the group (44).

Most of the available commercial fortifiers contain varying amounts of protein, carbohydrate, calcium, phosphate, other minerals, trace elements (zinc, manganese, magnesium, copper), vitamins, and electrolytes and are defined as “multi-nutrient HM fortifiers” (see Current Human Milk Fortifiers and Supplements) (43).

In an attempt to clarify the terminology regarding HM fortification practices, in 2010, World Association of Perinatal Medicine (WAPM) Working Group on Nutrition defined the fortification methods in current practice as follows (43):

1. Standard (STD) HM fortification
2. Individualized HM fortification:
  - Adjustable (ADJ) HM fortification (74, 77, 78)
  - Targeted HM fortification (76, 79–81)

The EMBA Working Group on HM Fortification adopts this terminology and Table 5 summarizes the characteristics of these methods.

**Table 5:** Current human milk fortification methods (43, 74, 76–79).

**Fortification method**	**Principle**	**Advantages disadvantages**
1. Standard (STD) Fortification	Fortification method currently in use in most of the neonatal units. A fixed amount of fortifier is added to a fixed volume of HM according to the manufacturers' instructions.	Practical. But has not solved the problem of protein undernutrition for VLBW infants. Despite STD fortification many VLBW infants continue to have suboptimal growth.
2. Individualized HM Fortification Methods a. Adjustable (ADJ) Fortification b. Targeted Fortification	Protein adequacy is monitored by BUN twice weekly, cut-off levels of BUN are 10–16 mg/dl^*^. If the level is <10 mg/dl extra protein is added to the STD fortification. Macronutrient concentrations in HM are analyzed and based on the results milk is supplemented with extra protein and/or fat.	Practical, not labor intensive. Doesn't need expensive devices. Monitors protein intake of each infant. Safeguards also against excessive protein intake. Proven to be effective in optimizing growth and protein intake with a RCT. A real individualization method taking into consideration each infant's protein requirement. All macronutrients can be supplemented. Bedside HM analyzers are required. May be labor intensive. Supplementation is done according to the population recommendations, does not take into consideration that each individual infant's requirement may be different.

*HM, human milk; VLBW, very low birth weight; BUN, blood urea nitrogen; RCT, randomized controlled trial*.

* *BUN levels of 10–16 mg/dl correspond to blood urea concentrations of 21.40–34.24 mg/dl (3.57–5.71 mmol/l)*.

## Standard (Std) Fortification

This is the most widely used fortification method. The standard practice is to add a fixed amount of multinutrient fortifier per 100 ml of HM to achieve the recommended nutrient intakes. This fixed amount has been calculated and determined by the manufacturer assuming a fixed protein content for all milk samples without considering intra-, inter-individual and temporal variations. Standard fortification is initiated usually when the fed milk volume is 50–100 ml/kg. Milan EMBA/ESPGHAN/AAP Joint Meeting Consensus recommends fortifying HM for preterm infants with a birthweight <1,800 g (42).

The updated Cochrane review (82) addressed the impact of STD multi-nutrient fortification of HM on growth, development, feeding tolerance and NEC in preterm infants. The systematic review evaluated 1,071 infants in 14 trials. The trials were generally small and weak methodologically. Meta-analyses provided low-quality evidence that STD multi-nutrient fortification of HM, in comparison to the unfortified HM, improved in-hospital weight gain, linear growth, and head circumference growth. Only very little data were available for growth and developmental outcomes beyond infancy and these did not show long-term advantage.

However, when comparisons are made between fortified HM in STD fashion and preterm formula (PF) (83–85) the findings indicate that despite fortification, HM fed preterm infants continue to grow more slowly than PF fed infants. Henriksen et al. (86) reported that 58% of VLBW infants fed predominantly fortified HM had EUGR at discharge. Maas et al. (87) evaluated in-hospital growth of 206 very preterm infants and found that standard deviation score for weight from birth to day 28 decreased more in infants with a cumulative milk intake >75% of all enteral feeds compared to those <25% HM intake. The trend toward poorer weight gain with higher proportions of HM intake persisted also at the time of discharge. Of course these findings cannot be a reason to favor preterm formula vs. HM to promote growth of VLBW infants. Considering all the clinical benefits deriving from the use of HM as already stated in the previous Sections, fortified HM should be the first feeding option for these infants. However, HM fortification should be optimized.

### Shortfalls With “Standard Fortification”

The reasons for the limited success with STD fortification include:

Undernutrition, particularly protein undernutrition: STD fortification does not take into account the variability of HM macronutrient content and variability of the infants' requirements. Preterm infants fed fortified HM in STD fashion receive less protein than they need due to “customary assumptions” as explained in the following paragraph. Protein is essential for tissue and organ development, and is a rate limiting factor for growth. A rate of postnatal growth similar to the intrauterine growth can be reached only with adequate protein and energy intakes (3.5–4.5 g/kg/d, 110–130 kcal/kg/d, respectively, Table 3). Standard fortification usually provides the recommended energy intakes, but cannot provide the adequate protein intakes for many VLBW infants (actual protein intake 2.8–2.9 g/kg/day) (88). Arslanoglu et al. (88) compared the assumed protein content of fortified HM samples and derived protein intakes to actual (measured) protein content/ intakes in a group of preterm infants. Actual protein intakes were consistently and significantly lower than assumed when fortification was performed in STD fashion (range of discrepancy between 0.5 and 0.8 g/kg/day). On the other hand, the differences in energy intake were small and not consistently significant. This observation was important, because it provided a rational basis for simply adding more protein to milk in those infants whose enteral diet came from milk, especially over long periods after birth (89). Similar findings have been reported in the following years by other researchers (75, 90, 91). Picaud et al. (75) showed that one third of extremely low birth weight infants (ELBW) infants needed supplementary protein to reach the expected weight gain. In the recent systematic review and meta-analysis regarding the macronutrient and energy composition of preterm human milk, Mimouni et al. (92) stated that protein content decreased massively (by one-half) and significantly from day 1–3 at week 10–12. During the same time frame; fat, lactose and energy content showed a significant linear increase. Very recently in PREMATURE MILK study Maly et al. (91) reported that protein content decreased during the first 3 weeks of lactation and the recommended protein intakes couldn't be reached with STD fortification in the majority of the infants.

The main reason for ongoing protein undernutrition despite HM fortification is that the STD regimen is based on assumptions about the protein content of the milk. Usually the assumed protein concentration by the manufacturers is 1.4–1.5 g/dl which only occurs during the first 2–3 weeks of lactation. HM protein concentration decreases with the duration of lactation and drops to around 1 g/dl by week 4–6 (43, 91). Thus, the protein intake would be inadequate most of the time throughout the fortification period (43, 44, 93).

Optimization of HM fortification is being widely studied. Improvement of the quality and source of the fortifiers, increasing the protein content of the products, early initiation of fortification are all efforts to improve STD fortification. An attempt at earlier initiation of fortification has resulted in better in-hospital head growth and weight gain in a very recent pre-, post- implementation study (94). However, a systematic review and meta-analysis aiming to ascertain whether randomized controlled trials determined the efficacy of early vs. late initiation of fortification on clinical outcomes gave inconclusive results. In this review Mimouni et al. (95) concluded that there is little evidence that early introduction of human milk fortification affects important outcomes.

Individualization of fortification is believed to be a solution to the problem of protein undernutrition with STD fortification and is currently the recommended method by scientific authorities and expert panels (29, 42, 43). The two methods of individualized fortification (Table 5); Adjustable and Targeted methods are discussed in the following Sections separately.

## Individualized Fortification

### Adjustable (ADJ) Fortification

ADJ method was designed specifically to avoid both protein undernutition and overnutrition. With this method, protein intake is adjusted on the basis of each infant's metabolic response. Human milk fortification is initiated with a multi-nutrient fortifier in a STD fashion and as soon as full strength fortification is tolerated, it is guided by blood urea nitrogen (BUN) levels as a surrogate for assessing protein adequacy. If BUN level is below a pre-defined threshold value (<10 mg/dl according to 2012 protocol) (77), extra protein is added in the form of protein supplement. If BUN level is above a specified value suggesting excessive protein (>16 mg/dl), the level of fortification is reduced (Tables 6, 7) (74, 77).

**Table 6:** The products required and the threshold values of the metabolic marker used for the Adjustable (ADJ) fortification method (77).

**Fortifier/supplement required**	
1. A multi-nutrient fortifier	
2. A protein supplement	
**Metabolic marker and threshold values used to adjust protein supply**	
Blood urea nitrogen (BUN)	<10 mg/dl–increase the fortification to the next level 10–16 mg/dl–no change
	>16 mg/dl–decrease the fortification by one level

**Table 7:** The scheme for adjustable fortification (updated in 2012) (77).

**Fortifier/supplement**	**Fortification levels and the amount of fortifier/supplement to be added (g per 100 ml HM)**					
	**−2**	**−1**	**0 Standard (STD)**	**+1**	**+2**	**+3**
Multi-nutrient HM fortifier	1/4 strength	Half strength	Full strength	Full strength	Full strength	Full strength
Protein supplement	−	−	−	0.4	0.8	1.2

This model was evaluated in a randomized controlled trial (RCT) by Arslanoglu et al. (74) and was found practical, feasible and effective to provide the preterm infants with adequate protein intakes approximating intrauterine protein intakes and better in-hospital growth compared to STD fortification. In this study the mean actual (measured) protein intakes reached 3.5 g/kg/d in ADJ group in the second week of the study, while it remained 2.8–2.9 g/kg/d in the STD group. During the 3 weeks intervention period the infants in ADJ group had better weight and head circumference gains compared to STD group (17 vs. 14 g/kg/d and 1.0 vs. 0.7 cm/wk, respectively).

ADJ fortification; i.e., adding extra protein on the basis of BUN measurements, has been in use since this publication with the protocol being refined in 2012 (77).

### The Updated Protocol for ADJ Fortification

The threshold range of BUN used to adjust protein supply was selected arbitrarily in the first study (9–14 mg/dl) (74). Adjusting the protein intake according to these values (74), the investigators observed that there was the need to increase the level of fortification during most of the fortification period; and the protein intakes could not reach the recommended intakes at the first week of the fortification. There was need to refine the protocol, and to be cautious only a small increase has been suggested. The threshold values for BUN were modified as 10–16 mg/dl (77, 78). Tables 6, 7 show the details of the current ADJ fortification regimen.

ADJ fortification starts as STD fortification when the fed milk volume reaches 50–80 ml/kg/d with multi-nutrient fortifier (Level 0). Protein adequacy is evaluated by twice weekly BUN determinations. Extra-protein is added in the form of protein supplement according to the protocol in 3 levels up to 1.2 g per 100 ml of HM (Table 7).

In 2013, Alan et al. (96) utilized a slightly modified form of ADJ fortification in their observational study and compared protein intakes and growth in VLBW infants fed HM fortified according to ADJ regimen to those fed in STD fashion (historical controls). The study replicated similar results in terms of higher protein intake and better in-hospital growth including linear growth with ADJ fortification.

Picaud et al. (75) in their recent retrospective study conducted on the preterm infants weighing <1,250 g at birth reported that 1/3 of extremely low birth weight infants required additional protein to supplement the standard fortification to achieve satisfactory weight gain. According to the practice in their NICU they used weekly measured urea levels and growth together to determine the need for extra protein. They confirmed the findings of Arslanoglu et al. (74) that extra protein supplementation not only improved weight gain but also head circumference gain.

In two observational studies, slightly modified forms of ADJ fortification were associated with both better growth and better neurodevelopmental outcomes. Ergenekon et al. in their retrospective study (97) reported better head growth and weight gain in NICU with ADJ fortification in very preterm infants. This improvement in growth was associated with significant improvement of Bayley scores at 18 months corrected age. Also, in the observational study of Biasini et al. (98), the improved growth with higher protein intakes in ELBW infants was associated with better neurodevelopment evaluated by Griffiths Mental Development Scores at corrected 12 months of age. At 24 months, small for gestational age (SGA) preterm infants having higher protein intake had higher scores.

Very recently, Mathes et al. (99) showed a highly positive correlation between plasma urea concentrations and actual protein intakes and urinary urea-creatinine ratio. They suggest that urinary urea-creatinine ratio, just like plasma urea concentrations may help to estimate the actual protein supply in preterm infants.

## Targeted Fortification

The concept of targeted fortification is to analyze macronutrient composition of HM and to fortify it in such a way that each infant always receives the amount of nutrient that is suggested in population-based recommendations. This method was proposed and studied first by Polberger et al. in 1999 (79) named as “individualized protein fortification of HM.” In this study, protein was the only nutrient considered for supplementation in addition to STD fortification. The milk was analyzed periodically and a target nutrient intake (protein) was delivered, which was 3.5 g/kg/day.

Parallel to the introduction of bedside human milk analyzers it has become possible for the researchers and neonatologists to analyze and tailor the macronutrient content based on real-time analysis of HM. In an observational study de Halleux et al. (81) compared standard vs. targeted (mentioned as “individualized” by the authors) fortification approaches; daily breastmilk composition was measured with a mid-infrared milk analyzer. They added modular fat to HM to reach a target fat content of 4 g/dl. A fortifier was added to reach a protein intake of 4.3 g/kg/day. As a result, the variability of macronutrients in the individualized approach was significantly decreased, but the average fat intake was 8.6 g/kg/day which exceeded the recommendations (see Table 3). Weight gain was superior to the STD fortification group and was similar to the formula fed group. Data regarding head circumference gain and linear growth were not shown.

Using a different approach, Hair et al. (59) in a two-center RCT measured breast milk energy density with a near-infrared analyzer. Infants received HM derived cream in addition to HM derived fortifier if energy density was <20 kcal/oz (67 kcal/100 mL). The HM derived cream was standardized to 25% lipids and contained 2.5 kcal/ml. Infants randomized to the HM derived cream group showed superior weight and length gain vs. the control group without cream. However, the validation studies with infrared analyzers have determined that the measurement of calories is not precise because of the inability to accurately measure lactose with these devices (80).

A pilot study conducted by Rochow et al. (76) has been the first to show the feasibility of targeted fortification of all macronutrients through twice daily breast milk analysis (near-infrared), using modular products to bring levels of fat to 4.4 g/dl, protein to 3 g/dl and carbohydrates to 8.8 g/dl. Matched pair analysis of 20 infants fed STD fortified milk was performed. Growth rates of the infants with targeted fortification were similar to the group with STD fortification (~20 g/kg/d). However, the authors showed a high correlation between volume of fed HM and weight gain only in the targeted group. They calculated an additional workload of 5–10 min per milk batch. The similar growth rate could be due to the fact that the STD group had higher milk intake than the targeted group (155 + 5 vs. 147 + 5 ml/kg/d). Another limitation to be improved was the 24 h delay between the milk analysis and the addition of the macronutrients.

Targeted fortification requires a milk analyzer, which is an expensive device, requiring careful calibration. Fusch et al. (100) draw attention to the need of recalibration of these analyzers since they were originally developed for use in the dairy industry and HM has a different matrix and optical characteristics from cow's milk. They conclude infrared analysis seems to be a promising tool for fat and protein with calibration, but lactose and therefore energy cannot be assessed with the current state of technologies.

Buffin et al. (101) compared fat and protein concentrations using two infrared analyzers and reference laboratory methods indicating the same important finding that bedside HM analyzers require recalibration before their use in practice.

In a recent RCT comparing targeted fortification to standard, McLeod et al. (102) did not find any improvement in growth and nutrition in a group of preterm infants born below 30 weeks of gestation. Interestingly, mean measured protein content in the targeted group was higher than the assumed value (1.6 vs. 1.4 g/100 ml), leading to lower amounts of fortifier added to the milk in the intervention group. The authors concluded that targeting fortification on measured composition is labor intensive requiring frequent milk sampling and precision measuring equipment.

## Post Discharge Fortification

There is no consensus about post discharge nutrition, however there is a position paper from ESPGHAN (103) and recent reviews (104–106), including one focusing on HM supplementation (106). The ESPGHAN position paper evaluated randomized trials published before 2004 and proposed fortifying HM up to at least 40 and possibly up to 52 weeks postconceptional age when infants were small for gestational age at discharge. However, the definition of being small for gestational age was not presented: bodyweight at discharge below 10th (moderate growth restriction) or 3rd percentile (severe growth restriction)? Meta-analysis and reviews suggested that there was evidence to use enriched nutrition [preterm formula (PF) rather than post discharge formula (PDF)] after discharge for formula fed babies. But this evidence was not strong enough to recommend fortification of HM after discharge (105, 106).

The last decade was marked by two trends. Firstly, a decrease in the incidence of extra-uterine growth restriction (16, 17, 107). Secondly, an increase in breastfeeding rates at discharge in these infants, despite significant heterogeneity between different neonatal units suggesting that there is room for improvement (108). Post discharge studies comparing enriched vs. standard nutrition highlighted the ability of some preterm infants -like term counterparts- to regulate their intake volume to compensate for differences in energy density between formulas (109–111). However, this is only true for preterm babies reaching term due date and beyond as it has been reported that many less mature preterm babies are not able to compensate for a low nutrient intake feed due to immature feeding skills (112). Therefore, there is a window of opportunity to optimize nutrition post discharge, which might explain why few studies reported a benefit for growth and mineralization of enriched post discharge nutrition.

Despite the widespread use of human milk fortifiers (HMF) for preterm babies on neonatal units there has been little reporting of its use post discharge. It might seem best practice to mirror the principle behind the use of post discharge formulas for formula fed babies, i.e., the bridge between a nutrient dense milk to one of lower density. In accordance with this there have been recommendations that fortifier is continued in preterm breast fed babies either to term or around 52 weeks post conceptional according to their growth trajectory (103, 113). Although the practicality of putting this into practice has been questioned by some reviewers because of the availability of HMF in different countries and the perceived, but not proven, problems with practicability (106), evidence is accruing that it is possible using many different methods (114).

It is known that babies exclusively breastfed post discharge can have reduced bone mineral density and lower lean body mass than formula fed babies (115), although this may have improved with more recent feeding practices on neonatal units. But it does suggest that some fortification of breast milk post discharge will help nutritional status as well as growth.

There have been 3 reports of randomized controlled trials fortifying breast milk post discharge, to 4 months corrected age (116), for around 5–6 months after discharge (117), and to around 12 weeks after discharge (118). Two used commercial HM fortifiers (116, 118) and one (117) a powdered preterm formula. Table 8 shows the nutrient intervention and numbers of infants in these trials. No difference in growth was found by Zachariassen et al. (116). However, this group did find better lung function in the fortification group at 6 years old (119). O'Connor et al. (118) found better weight, length and bone mineral density and better head growth in babies <1,250 g birth weight, all of which were maintained to 1 year (120). There is also evidence of better visual function (121). Neurodevelopmental outcome at 12–18 months was not found to be different between groups (117, 120). In these studies there may not have been sufficient protein given to achieve an optimal growth and possibly neurodevelopmental outcome. In contrast energy should not be a limiting nutrient for a baby feeding fully responsively at the breast as they will access all the energy rich hind milk they require.

**Table 8:** Nutrient interventions in the randomized controlled trials addressing the effects of fortifying human milk post discharge (116–118).

	**O'Connor et al. (118)**	**Zachariassen et al. (116)**	**Da Cunha et al. (117)**
Additional Protein (g)	0.8/kg	1.37/day	0.5/day
Additional energy (kcal)	~10–15/kg	17/day	20/day
Numbers assessed–intervention	19	102	26
Numbers assessed-Control	20	105	27
Outcomes	Growth at 4,8,12 weeks after discharge Energy and some nutrient intakes (diaries)	Growth at 2,4,6,12 months corrected age Blood urea nitrogen, phosphorus, hemoglobin levels	Neurodevelopment assessed by Bayley III Scale at 12 months corrected age

All studies found the HMF to be well-tolerated however each used a different method to administer the fortifier, one adding the entire dose into one bottle per day (116) with no adverse gastrointestinal symptoms reported. Another was by bottle but given spread out over the day (117) and the third by cup twice a day (118). None reported any adverse effect on breast feeding rates in the fortified group. A finger feeding device to administer fortifier has recently been evaluated and was well-accepted (122). Technical improvements are required and a large randomized study will be necessary to evaluate the benefits of such a strategy.

There are 2 reports looking at the effect of fortifier post discharge on breastfeeding. One is a case study where breastfeeding was maintained by the addition of fortifier post discharge rather than formula in a baby failing to thrive on the breast (123). A second suggests an improvement in breastfeeding rates at 6 weeks post conceptional age in a group of babies discharged on fortifier compared to a group on breast milk alone (124).

In theory, a gradual step down from full fortification would seem appropriate for breastfed babies to allow adaption to the lower nutrient intake of unfortified breast milk and to support the rapid growth that occurs around term due date.

As studies that evaluated post discharge HM fortification showed no deleterious effect on breastfeeding rates, it is proposed that HMF post discharge is considered in breastfed babies where a post discharge formula would be used were they formula fed, particularly if they have not grown well while on the neonatal unit. One group who might benefit being babies with bronchopulmonary dysplasia BPD (125). More work is needed to assess an optimal amount, length of time and method of administering fortifier in a breast feeding baby post discharge.

## Conclusions-Comments, Recommendations

**Conclusions-Comments**

- Evidence indicates that HM is the best nutrient uniquely suited not only to term but also to preterm infants conferring health benefits at short and long-term including protection against NICU challenges such as NEC, ROP, BPD sepsis and neurocognitive improvement. Therefore, it is the first choice in preterm feeding.
- Unfortified HM doesn't provide sufficient amounts of nutrients to tiny preterm infants when fed at usual feeding volumes. To prevent EUGR which is associated with poor neurocognitive outcome and to avoid specific nutrient deficiencies, nutrient fortification of HM is necessary.
- The fortification methods in current use are: **1**. Standard fortification, **2. I**ndividualized fortification: “Adjustable fortification” and “Targeted fortification.”
- Despite STD fortification many VLBW infants continue to have suboptimal growth. Optimization of HM fortification is necessary.
- ADJ fortification has been shown to improve protein intakes, somatic and head growth and seems to be a practical method to optimize HM fortification.
- Targeted fortification, being feasible and effective in some trials, needs to be improved.
- Improvement of the quality of HMF is another important issue. Although HM-based fortifier seemed to be promising and some studies suggested a benefit in terms of morbidity and mortality when babies are fed an exclusively human milk based diet using these products, there are still concerns about the efficacy, safety and ethical issues.
- There is no strong evidence to support the use of hydrolyzed protein source in fortifiers.
- There is no consensus about post discharge nutrition. Studies that evaluated post discharge HM fortification showed no deleterious effect on breastfeeding rates, and suggested some advantages.

**Recommendations**

- Given the solid evidence, HM feeding has become a basic right for preterm infants. Mother's own milk is the first choice in preterm infant feeding and strong efforts should be made to promote lactation. When mother's milk is not available, donor human milk is the best alternative.
- EMBA WG on HM Fortification, in parallel with Milan Consensus (42) recommends fortification of HM for preterm infants with a birthweight <1,800 g.
- Human milk fortification can be started safely with multi-nutrient fortifiers when the milk volume reaches 50–80 ml/kg/d.
- Optimization of HM fortification is required. Individualized fortification (Adjustable or Targeted) is the recommended method for HM fortification. Targeted Fortification may need some fine tuning.
- Quality improvement of the fortifiers is an ongoing process. Because of the limited efficacy and safety data and ethical concerns, it is too early to draw conclusions about the use of HM-based fortifiers.

**FUTURE RESEARCH DIRECTIONS**

- Research addressing the nutritional management in specific groups of preterm infants (such as BPD, IUGR)
- Randomized controlled trials assessing the efficacy and safety of HM fortification after discharge in different groups depending on their status at discharge
- Randomized trials comparing the efficacy and safety of ADJ vs. Targeted Fortification
- Defining the reasonable and replicable study endpoints including neurocognitive outcomes, body composition in large cohorts
- Optimization of the quality of fortifiers (amount and quality of protein, source of energy, EFA content) while considering ethical dilemmas

## Author Contributions

SA is leading the EMBA WG on Human Milk Fortification. J-CP, C-YB, CK, DL, and PT are the components of the WG. All contributed to the construction and writing of the manuscript. GM, EB, GW, AW, AGa, AGr, CG, and DB commented on the manuscript as the components of the EMBA Board.

### Conflict of Interest Statement

The authors declare that the research was conducted in the absence of any commercial or financial relationships that could be construed as a potential conflict of interest.
